# Control of Pim2 kinase stability and expression in transformed human haematopoietic cells

**DOI:** 10.1042/BSR20150217

**Published:** 2015-12-08

**Authors:** Kévin Adam, Mireille Lambert, Elsa Lestang, Gabriel Champenois, Isabelle Dusanter-Fourt, Jérôme Tamburini, Didier Bouscary, Catherine Lacombe, Yael Zermati, Patrick Mayeux

**Affiliations:** * Institut Cochin, INSERM U1016, CNRS UMR 8104, Université Paris Descartes, Sorbonne-Paris-Cité, Paris 75014, France; † Ligue Nationale Contre le Cancer, équipe labellisée, Paris 75014, France; ‡ Hopital Cochin, AP-HP, Paris 75014, France

**Keywords:** leukaemia, multiple myeloma, proteasome, protein degradation, ubiquitin

## Abstract

The oncogenic Pim2 kinase is overexpressed in several haematological malignancies, such as multiple myeloma and acute myeloid leukaemia (AML), and constitutes a strong therapeutic target candidate. Like other Pim kinases, Pim2 is constitutively active and is believed to be essentially regulated through its accumulation. We show that in leukaemic cells, the three Pim2 isoforms have dramatically short half-lives although the longer isoform is significantly more stable than the shorter isoforms. All isoforms present a cytoplasmic localization and their degradation was neither modified by broad-spectrum kinase or phosphatase inhibitors such as staurosporine or okadaic acid nor by specific inhibition of several intracellular signalling pathways including Erk, Akt and mTORC1. Pim2 degradation was inhibited by proteasome inhibitors but Pim2 ubiquitination was not detected even by blocking both proteasome activity and protein de-ubiquitinases (DUBs). Moreover, Pyr41, an ubiquitin-activating enzyme (E1) inhibitor, did not stabilize Pim2, strongly suggesting that Pim2 was degraded by the proteasome without ubiquitination. In agreement, we observed that purified 20S proteasome particles could degrade Pim2 molecule *in vitro*. Pim2 mRNA accumulation in UT7 cells was controlled by erythropoietin (Epo) through STAT5 transcription factors. In contrast, the translation of Pim2 mRNA was not regulated by mTORC1. Overall, our results suggest that Pim2 is only controlled by its mRNA accumulation level. Catalytically active Pim2 accumulated in proteasome inhibitor-treated myeloma cells. We show that Pim2 inhibitors and proteasome inhibitors, such as bortezomib, have additive effects to inhibit the growth of myeloma cells, suggesting that Pim2 could be an interesting target for the treatment of multiple myeloma.

## Abstract (precis)

The constitutively-active kinase Pim2 overexpressed in transformed cells constitutes an attractive target in cancer. We show that both Pim2 degradation realized by the proteasome in an ubiquitin-independent manner and Pim2 translation are constitutive.

## INTRODUCTION

The Pim family of serine/threonine kinases consists of three members, Pim1, Pim2 and Pim3 that play important roles in cancer. Pim1 and Pim2 proto-oncogenes were first identified as provirus integration sites for Moloney murine leukaemia virus and the resulting overexpression of these kinases leads to lymphoma and leukaemia in mice. Pim3 was next identified by sequence homology with Pim1 and Pim2. Pim kinases are frequently overexpressed in human cancer and especially in haematological malignancies where they support malignant cell proliferation and survival [1]. The Pim kinases have therefore emerged as potential key drug targets in many human cancers and several inhibitors have been recently described that are promising as suggested by their demonstrated efficiency in pre-clinical models of acute myeloid leukaemia (AML) [2,3]. Pim1 and Pim2 are most frequently involved in haematological malignancies whereas Pim3 is more often de-regulated in solid tumours [4]. Several reports have shown that Pim2 is strongly involved in the survival and proliferation of myeloma cells suggesting that it could constitute an interesting therapeutic target in this disease [5–7]. We have shown that Pim2 was constantly expressed in primary blasts from AML. In these cells, Pim2 is involved in protein translation by controlling 4E-BP1 phosphorylation. Pim1 that is less frequently expressed than Pim2 in AML cells does not correct Pim2 knockdown in these cells [8]. Several clinical trials are currently ongoing to test the potential of Pim kinase inhibitors in multiple myeloma, AML and myelodysplastic syndromes (https://clinicaltrials.gov). Thus, the understanding of the mechanisms that control the activity of Pim2 kinase is of utmost importance for cancer therapy.

The overall structure of the Pim kinases is simple since these proteins are almost restricted to the kinase domain with short sequences both before and after this domain. Moreover, in contrast with most other kinases that need to be phosphorylated in the activation loop (A-loop) to be active, Pim kinases constitutively adopt an active conformation due to the presence of an acidic residue in the A-loop (Asp^200^ in Pim1, Asp^196^ and possibly Asp^198^ in Pim2) that forms a salt bridge with basic residues of the catalytic loop (C-loop) and thus mimics the phosphorylable serine or threonine residue that is present in other kinases [9,10]. As a result, Pim kinases are constitutively active and essentially regulated at the level of protein expression and stability. Pim1 gene expression is regulated by STAT5. Moreover, Pim1 mRNA presents structured 5′- and 3′-ends and its translation is also regulated [11]. The Pim kinase proteins appear to be rather unstable and are degraded by the proteasome. Nevertheless, to the best of our knowledge, no ubiquitin ligase regulating their degradation has been identified and the mechanisms that control their stability remain unclear. Pim1 degradation seems to be controlled by a phosphorylation/de-phosphorylation mechanism that involves the protein phosphatase PP2A, SOCS1 and the proteasome [12]. Pim1 was found to bind to the heat-shock proteins (HSPs), HSP90 and HSP70, which regulate its stability. However, whereas HSP90 stabilizes Pim1, HSP70 promotes its degradation by the proteasome [13]. The mechanisms leading to the divergent effects of these chaperon proteins regarding Pim1 are not understood. A recent report shows that the translationally-controlled tumour protein (TCTP/TPT1) associates with the C-terminus region of Pim3 and protect Pim3 from proteasomal degradation leading to enhanced tumour pancreatic cell survival and proliferation [14]. The regulation of Pim2 stability and expression is less documented and many features of Pim1 have been frequently extended to Pim2 without the careful verification that the mechanisms that control the two kinases are indeed identical. Pim1 and Pim2 exhibit 61% of similarity only that is restricted to the kinase domain; the small sequences surrounding the catalytic domain being fully divergent [15]. Three isoforms of Pim2 resulting from the use of three translation start sites are produced from the same mRNA in murine cells. Isoforms 1 (40 kDa) and 2 (37 kDa) use non-classical CUG translation initiation codons whereas the first AUG codon that gives rise to isoform 3 (34 kDa) is not embedded in a Kozak sequence and is translated with a low efficiency [16]. Two isoforms of Pim2 only were frequently reported in human cells although mRNA sequences present a high homology between human and mouse [3,17,18].

In the present study, we address the question of the mechanisms that control Pim2 stability. We show that transformed haematopoietic human cells, either of lymphoid or myeloid origin, express three isoforms of Pim2. The relative expression of the three isoforms was identical in all tested cells. All isoforms showed dramatically short half-life although the longer isoform was significantly more stable than the others demonstrating that the N-terminus of the protein participates to the regulation of its stability. Although Pim2 degradation was mainly realized by the proteasome, our results show that its degradation did not require its ubiquitination. Blocking the proteasome activity led to a rapid and dramatic accumulation of the active kinase. This effect was observed in myeloma cells treated with the proteasome inhibitor bortezomib that is used for the treatment of multiple myeloma. We show that blocking Pim kinase activity strongly suppressed the growth of myeloma cells and that proteasome and Pim kinase inhibitors presented additive inhibitory effects in these cells.

## MATERIALS AND METHODS

### Cells and general experiment design

Human myeloma cells (RPMI8226, U266 and AMO1) were cultured in Roswell Park Memorial Institute medium (RPMI) 1640 medium supplemented with 10% fetal calf serum (FCS), as previously described [19]. The AMO1 cell line was a kind gift of Dr M. Amiot (University of Nantes). Human AML cells (MV4.11, Molm14 and UT7) were cultured in alpha Minimum Essential Medium (α-MEM) supplemented with 10% FCS and 2 units/ml recombinant Epo for UT7 cells as described [20,21]. All cells were maintained and used for experiments in exponentially growing phase. Unless otherwise indicated, experiments using UT7 cells were performed in the presence of 2 units/ml Epo to maintain a high level of Pim2 expression. However, for some experiments (see ‘Results’) UT7 cells were Epo-deprived by incubating cells for 18 h in Iscove Dulbecco's Modified Eagle's medium (DMEM) medium supplemented with 0.4% BSA and 50 μg/ml iron-loaded transferrin. Each reported experiment was performed at least three times with similar results and either a representative experiment or mean ± S.D. of all the performed experiments are reported.

### Lentiviral transductions

Lentiviral vectors encoding GFP and shRNA against luciferase (control shRNA), STAT5A, STAT5B or STAT5A/B were produced as described [22]. Lentiviral particles were added to the indicated cells in order to get at least 95% of transduction, as detected by GFP flow cytometry.

### Antibodies and reagents

Antibodies used to detect Pim2 by western blot were from Cell Signaling Technology and those used for immunoprecipitation were laboratory made by immunizing rabbits with a bacterially expressed recombinant protein corresponding to the full short isoform of Pim2 fused to a His-tag. Anti-ubiquitin antibodies (FK2) and de-ubiquitinase (DUB) inhibitor WP1130 were from Viva Biosciences, anti-EpoR (Epo receptor) antibodies (C-20) were from Santa Cruz. Phospho-specific antibodies against STAT5, Bad, Erk and Akt were from Cell Signaling Technology. Rabbit antibodies against ubiquitin used for immunoprecipitation (Apu2) were from Millipore. The pan-Pim kinase inhibitor 5-[[2-[(3R)-3-aminopiperidin-1-yl]biphenyl-3-yl]methylidene]-1,3-thiazolidine-2,4-dione (AZD1208, [3]) and the Nedd8-activating enzyme inhibitor ((1S,2S,4R)-4-(4-(((S)-2,3-dihydro-1H-inden-1-yl)amino)-7H-pyrrolo[2,3-d]pyrimidin-7-yl)-2-hydroxycyclopentyl) methyl sulfamate (MLN4924) were from Active Biochem. Bortezomib was from Selleckchem, lactacystin, N-acetyl-L-leucyl-L-leucyl-L-norleucinal (LLnL), Akt inhibitor VIII and epoxomicin were from Calbiochem (Merck Millipore). All other reagents were purchased from Sigma–Aldrich.

### Western blot experiments

Western blot experiments were performed as previously described [20]. To increase the detection of ubiquitination forms, proteins were denatured on membranes using 6 M guanidium chloride after transfer [23]. Images were recorded using a Fuji LAS 3000 camera and analysed using the MultiGauge V3.00 software (FujiFilm).

### RNA extraction and reverse transcriptase quantitative PCR

Cells were lysed in Trizol reagent (Invitrogen) and total RNA was purified using QIAmp Mini Spin columns (Qiagen) following manufacturer's recommendations. Complementary DNAs were prepared from total RNA using the Maxima First Strand cDNA Synthesis kit for reverse transcription quantitative polymerase chain reaction (RT-qPCR) (ThermoScientific). Quantitative PCR was performed using the LightCycler 480 SyberGreen I Master kit (Roche) and the LightCycler 480 System (Roche). Three reference genes (β2 microglobulin, ubc and β-actin) were used for data normalization. The following primers were used: Pim2 (forward 5′-GCCTCACAGATCGACTCCAG-3′, reverse 5′-CTGAGTCTGACAAGGGGGAC-3′); ubc (forward 5′-ATTTGGGTCGCAGTTCTTG-3′, reverse 5′-TGCCTTGA-CATTCTCGATGGT-3′), β-actin (forward 5′-TCCCTGGA-GAAGAGCTACG-3′, reverse 5′-AGCACTGTGTTGGCGTA-CAG-3′), β2m (forward 5′-CCACTGAAAAAGATGAGT-ATGCCT-3′, reverse 5′-CAAACCTCCATGATGCTGAT-TACA-3′).

### Sub-cellular fractionation

To prepare cytosolic and nuclear fractions, washed cells were suspended in ice-cold hypotonic buffer (Tris/HCl 10 mM, pH 7.4, NaCl 10 mM, MgCl_2_ 3 mM, PMSF 0.5 mM, DTT 2 mM) and 0.2% NP40 were added. Nuclei were pelleted by centrifugation (400 ***g***, 4 min) and supernatants were centrifuged at 10000 ***g*** for 2 min to obtain the crude cytosolic fraction. Nuclei were washed with hypotonic buffer and solubilized in Laemmli sample buffer.

### *In vitro* degradation by 20S proteasome

Pim2 was partially purified from AMO1 cells that were treated for 1 h with 100 nM Bortezomib by chromatography using first a strong anion exchanger column (Resource Q, GE-Healthcare) and then a Superdex G200 size exclusion chromatography column (GE Healthcare). Fractions containing Pim2 were identified by western blot, pooled and concentrated using 10 kDa centrifuge concentrators (Millipore). Purified 20S proteasomes were obtained from VivaBioscience. Incubation buffer was Tris/HCl 50 mM, pH 7.5, containing 150 mM NaCl and 1 mM DTT. Hundred nanograms of purified 20S proteasome were incubated with 5 μg of protein from concentrated Superdex G200 fractions in a total volume of 20 μl. Incubation was ended by adding 20 μl of 2× electrophoresis sample buffer and boiling for 5 min.

### Kinase assay

Myeloma cells treated or not with Bortezomib were solubilized with solubilization buffer (Tris/HCl 10 nM, NaCl 150 mM, EDTA 5 mM, pH 7.4) containing protease (Complete™, Roche) and phosphatase (PhosStop, Roche) inhibitors and 1% NP40. Cell extracts were cleared by centrifugation and Pim2 was immunoprecipitated using laboratory-made antibodies and Protein G Sepharose beads (GE Healthcare). Immunoprecipitates were washed successively with solubilization buffer, PBS and kinase buffer (kinase buffer: HEPES 20 mM, MgCl_2_ 10 mM, DTT 1 mM, pH 7.4). Beads with immunoprecipitated Pim2 were incubated for 30 min at 30°C with kinase buffer containing 50 μM ATP, phosphatase inhibitors (Sigma-Aldrich P0044) and 500 ng of purified GST–Bad as substrate (Sigma–Aldrich). Since the molecular mass of GST–Bad (47 kDa) is close to that of IgG heavy chains, supernatants of the kinase assays were used for Bad phosphorylation analysis by western blot and beads were then eluted for Pim2 immunoprecipitation control by western blotting.

### Measurement of myeloma cell proliferation

Twenty thousand myeloma cells were plated with drugs in 96-well microplates in a total volume of 100 μl and incubated for 48 h. For each drug combination, triplicate samples were seeded and analysed. During the last 2 h of incubation, 10 μl of UptiBlue (Interchim) were added. Fluorescence was read using a Typhoon fluorescence scanner (GE-Healthcare) with excitation at 532 nm and recording using a 580BP30 filter. Fluorescence was quantified using the MultiGauge software. To determine whether drugs presented additive or synergistic activities, the Chou and Talalay method was used through the Compusyn software (http://www.combosyn.com) [24].

## RESULTS

### *Pim2* expression in haematopoietic cells

We tested Pim2 expression in three cell lines derived from AML cells (MOLM14, MV4.11 and UT-7) and in three myeloma-derived cell lines (AMO1, RPMI8226 and U266). In all these cells, we detected significant amounts of Pim2 protein that always presented three isoforms with constant relative amounts (Figure 1A). Pim2 isoform expression was quantified in three samples for each cell line: isoform 2 was always the most expressed whatever the cell type and accounted for 59±6% of Pim2 whereas isoform 1 (28±5%) and isoform 3 (13±4%) were less expressed. Previous reports only detected two Pim2 isoforms in human cells [17,18]. To control that the three bands observed in western blots indeed corresponded to Pim2, we used MOLM-14 cells expressing a doxycycline-inducible Pim2 shRNA [25]. As shown in Figure 1(B), the three bands disappeared in shRNA-transfected cells, confirming that, like murine cells, human leukaemic cells express three Pim2 isoforms with 27, 32 and 36 kDa apparent molecular masses. The structure of the three Pim2 isoforms according to Nawijn et al. [15] is presented in Figure 1(C). Calibration of the western blots using recombinant GST–Pim2 allowed to calculate that exponentially growing UT7 cells express approximately 40000 Pim2 molecules per cell (result not shown). The amounts of Pim2 in AML cells were 10-fold lower than those present in myeloma or UT7 erythroleukaemia cells.

**Figure 1:**
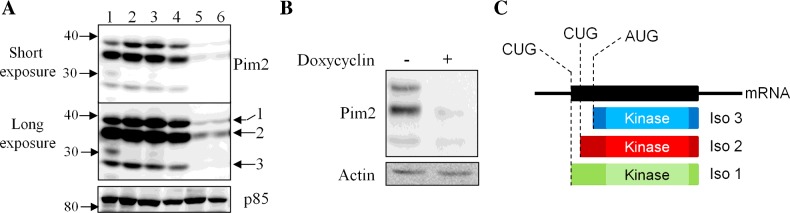
Pim2 expression in transformed haematopoietic cells (**A**) Exponentially growing myeloma cells [Amo1 (1), RPMI8226 (2) and U266 (3) and AML cells (UT-7; 4), Molm 14 (5) and MV4.11(6)] were analysed by western blotting using anti-Pim2 antibodies. A longer exposure of the blot is presented in the medium panel and the three Pim2 isoforms are labelled. The regulatory subunit of PI3K (p85) was used as loading control. (**B**) Molm 14 expressing a doxycycline-inducible Pim2 shRNA was used to verify the specificity of anti-Pim2 antibody. (**C**) Schematic structure of the three Pim2 isoforms.

We used the growth factor-dependent UT7 erythroleukaemia cell line to analyse the regulation of Pim2 expression. We observed that Pim2 expression was strongly decreased, although not totally abolished, in growth factor-deprived UT7 cells. Erythropoietin (Epo) stimulation increased Pim2 expression in these cells. In contrast, SCF (stem cell factor) or FCS did not modify Pim2 expression. Whereas both SCF and Epo stimulated Erk and Akt, only Epo activated STAT5 in UT7 cells (Figure 2A). To verify that Pim2 expression was indeed controlled by STAT5, STAT5A and/or STAT5B were knocked down using three different shRNA [22]. Figure 2(B) shows that knockdown of STAT5A and STAT5B reduced Pim2 expression to a level similar to that observed in growth factor-deprived cells. Lastly, we measured Pim2 mRNA expression in UT7 cells either growth factor deprived or stimulated by Epo or SCF and we observed that Epo but not SCF indeed regulated Pim2 mRNA expression (Figure 2C). Thus, Pim2 is regulated at the level of mRNA accumulation by extracellular signalling that activates STAT5.

**Figure 2:**
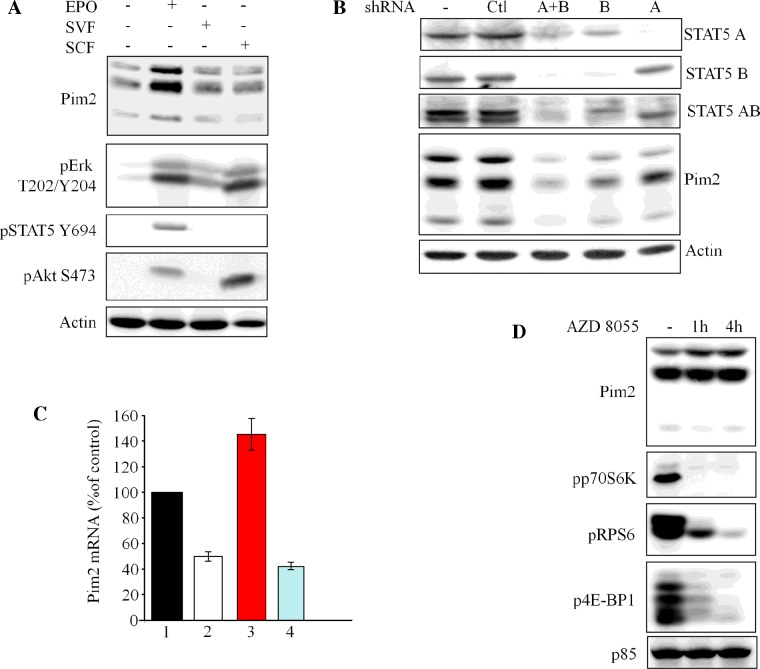
Pim2 transcription is controlled by STAT5 in UT7 cells (**A**) Epo stimulates Pim2 expression in UT7 cells. UT7 cells were incubated for 18 h with or without 2 units/ml Epo, 10% FCS (SFV) or 100 ng/ml SCF and cell extracts were analysed by western blots using anti-Pim2, anti-phospho-Erk, anti-phospho-STAT5 or anti-phospho-Akt antibodies. (**B**) STAT5 knockdown impairs Epo-stimulated Pim2 expression. Uninfected UT7 cells (–) or UT7 cells infected with lentiviral vectors encoding control shRNA (Crl) or shRNA targeting STAT5A (**A**), STAT5B (**B**) or both STAT5A and STAT5B mRNA (**A**+**B**) were incubated with 2 units/ml Epo and analysed by western blots for Pim2 protein expression. (**C**) Epo controls Pim2 mRNA accumulation. UT7 cells cultivated in the presence of 2 units/ml Epo (1) were Epo-deprived for 18 h (2) and re-stimulated for 6 h with 2 units/ml Epo (3) or 100 ng/ml SCF (4). Pim2 mRNA was quantified by quantitative RT-qPCR as described in the ‘Materials and Methods’ section. Each bar corresponds to the mean ± S.D. of three independent experiments. (**D)** Blocking mTORC1 pathway does not decrease Pim2 protein accumulation. Exponentially growing UT7 cells were incubated with 1 μM AZD8055 for the indicated times and analysed by western blot for Pim2 expression. Inhibition of mTORC1 was controlled using anti-phospho-P70S6K (Thr^389^), anti-phospho-RPS6 (Ser^235/236^) and anti-phospho-4E-BP1 (Thr^37/46^) antibodies.

Next, we tested whether Pim2 expression is controlled at the translation level by mTORC1. Epo-stimulated UT7 cells were treated by the mTOR catalytic inhibitor AZD 8055. Although a strong inhibition of the phosphorylation of p70S6 kinase, RPS6 and 4E-BP1 was observed, Pim2 expression was not modified (Figure 2D) even after 4 h of mTORC1 inhibition. Thus, Pim2 expression is not controlled at the translation level by mTORC1.

### Stability of Pim2 isoforms

We used cycloheximide to block protein synthesis in order to determine the stability of the Pim2 isoforms. All three isoforms exhibited dramatically short half-lives ranging from 9 and 16 min for isoforms 2 and 3 respectively and up to 1 h for isoform 1 (Figures 3A and 3B). The half-lives of the three isoforms were similar in all cell lines tested. Since the three isoforms originate from a single mRNA, their translation efficiency can be calculated by taking into account both their stability and their relative abundance. We calculated that isoforms 1 and 2 are translated 2- and 16-fold respectively more efficiently than isoform 3, although isoform 3 is translated from an AUG codon whereas isoforms 1 and 2 are translated from CUG codons. We tested whether the difference of stability of the three isoforms could be due to different sub-cellular localizations. Since no antibodies that are able to specifically detect each Pim2 isoform are available, we transfected human embryonic kidney (HEK) 293 cells with expression vectors encoding each Pim2 isoform. Figure 3(C) shows that all Pim2 isoforms were mostly cytoplasmic and that no significant difference of localization could be detected among the three isoforms. To verify these results on the sub-cellular localization of the endogenous proteins, nuclear and cytoplasmic fractions were prepared from UT7 cells and each fraction was analysed by western blot. Control proteins showed expected sub-cellular localization for the cytoplasmic p85 adaptor of the phosphoinositide 3-kinase (PI3K) molecule and the nuclear GATA1 transcription factor. The three Pim2 isoforms were exclusively found in the cytoplasmic fraction, confirming the immunofluorescence results (Figure 3D). Thus, the different stability of the three isoforms cannot be explained by different sub-cellular localizations.

**Figure 3:**
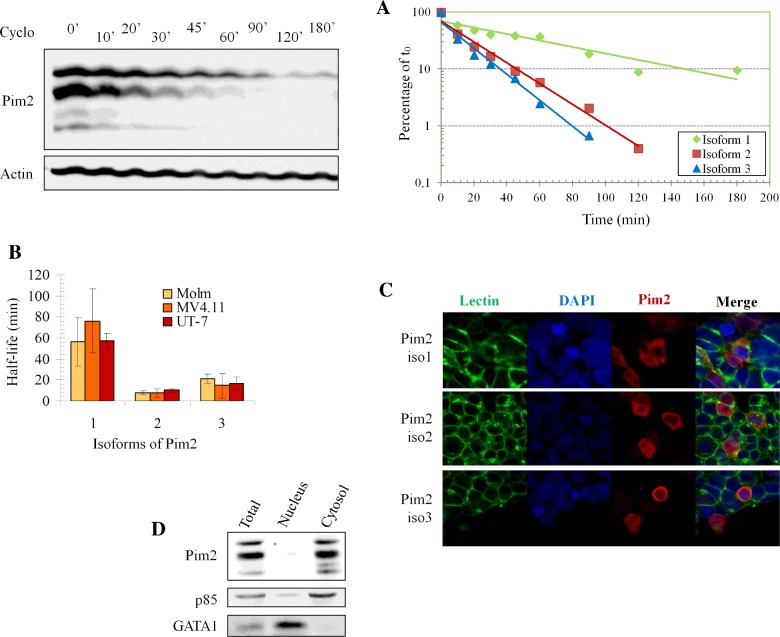
Stability of the Pim2 isoforms (**A** and **B**) The three Pim2 isoforms present significantly different stability. Exponentially growing UT7 cells were incubated with 200 μM cycloheximide for the indicated times and analysed by western blot using anti-Pim2 antibodies (left panel). Western blot results were analysed using the MultiGauge software. Densitometric values of each Pim2 isoforms were first normalized to the corresponding densitometric values of actin and expressed as the percentage of initial (t_0_) normalized value (right panel). A representative experiment is presented (**A**). Similar experiments were realized using Molm14 and MV4-11 cells. Error bars represent S.D.s of the three determinations (**B**). (**C** and **D**) The three Pim2 isoforms have similar cellular localization. HEK 293 cells were transiently transfected with expression plasmids encoding each Pim2 isoform and analysed by immunofluorescence. Wheat germ lectin, labelled with Alexa 488 and DAPI, were used to label the plasma membrane and the nucleus respectively (**C**). Sub-cellular localization of endogenous Pim2 was tested in UT7 cells as described in the ‘Materials and Methods’ section (left panel). PI3K (p85) and the transcription factor GATA1 were used to control the efficiency of the fractionation process (**D**).

### Pim2 degradation by the proteasome is not controlled by Erk, Akt or mTORC1 signalling pathways

We tested whether Pim2 proteins are degraded by the proteasome. Treating cells with different proteasome inhibitors such as LLnL or bortezomib always leads to stabilization of the three Pim2 isoforms (Figure 4A). We obtained similar results using two other proteasome inhibitors, lactacystin and epoxomicin (result not shown). Both AML and myeloma cells showed strong accumulations of Pim2 after treatment with proteasome inhibitors. Then, we tested the effects of blocking the main Epo-activated pathways in UT7 cells on Pim2 stability. To better identify a possible effect of these pathways on Pim2 degradation, we tested the effects of their inhibition in the presence of cycloheximide to block Pim2 synthesis. Blocking Erk and PI3K/mTOR pathways did not modify Pim2 stability. Moreover, neither the general kinase inhibitor staurosporine nor the okadaic acid, a PP1 and PP2A phosphatase inhibitor, noticeably affected the stability of any Pim2 isoform (Figure 4B). Altogether, these data show that Pim2 is rapidly degraded by the proteasome and suggest that this degradation is not controlled by phosphorylation/dephosphorylation mechanisms.

**Figure 4:**
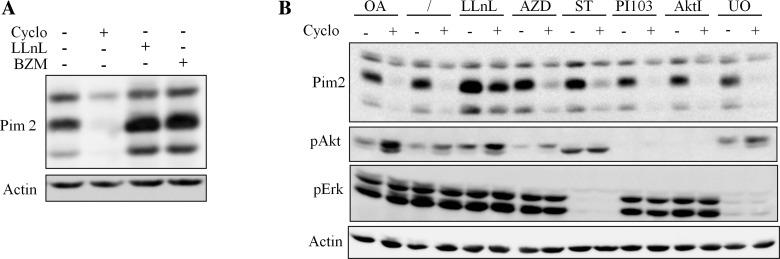
Regulation of Pim2 degradation by the proteasome (**A**) The three Pim2 isoforms are stabilized by proteasome inhibitors. RPMI 8226 myeloma cells were treated for 1 h with 50 μM LLnL, 100 nM bortezomib (BZM) or 200 μM cycloheximide and Pim2 expression was analysed by western blot. (**B**) The stability of Pim2 isoforms was not modified by kinase or phosphatase inhibitors. Exponentially growing UT7 cells were treated for 1 h with either 100 nM okadaic acid (OA), 50 μM LLnL, 4 μM AZD 1208, 1 μM staurosporine (ST), 1 μM PI103, 5 μM Akt inhibitor VIII (AktI) or 10 μM UO126. Cycloheximide (200 μM) or vehicle (0.1% ethanol) was added for the last 30 min of incubation. Inhibitor efficiency was controlled using antibodies against phosphorylated Akt (Ser^473^) and Erk.

### Pim2 could be degraded by the proteasome without ubiquitination

Although some proteins can be degraded by the proteasome without ubiquination [26], in most cases proteins need to be ubiquitinated prior to proteasome degradation. Many intracellular proteins are ubiquitinated by protein complexes that use cullins as scaffold proteins. Ubiquitin ligase activity of these complexes requires the neddylation of the cullin component [27]. MLN 4924 inhibits the NEDD8-activating enzyme leading to suppression of these ubiquitinating complexes. However, MLN 4924 did not stabilize any Pim2 isoforms in UT7 AML cells (Figure 5A). We controlled that MLN 4924 inhibited the degradation of the EpoR in the same UT7 cells (Figure 5B). Indeed, we have previously shown that the EpoR was degraded by the proteasome after ubiquitination by an ubiquitin ligase complex containing β-TRCP that uses cullin as scaffold protein [28]. Thus, we concluded that Pim2 is not targeted to proteasome degradation by a cullin-containing ubiquitin ligase complex.

**Figure 5:**
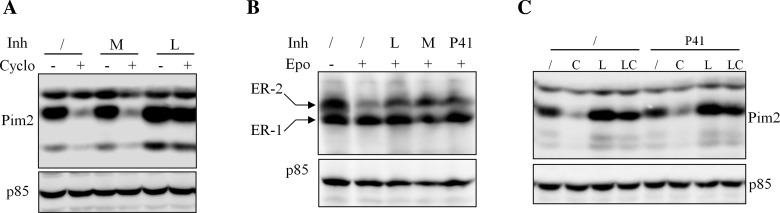
Ubiquitin ligase inhibitors did not modify Pim2 degradation (**A**) The neddylation inhibitor MNL4924 did not inhibit Pim2 degradation. Exponentially growing UT7 cells were incubated for 1 h with 1 μM MLN4924 or 50 μM LLnL. Cycloheximide (200 μM) or vehicle (0.1% ethanol) was added for the last 30 min of incubation and Pim2 expression was analysed by western blotting. (**B**) Control of ubiquitin ligase inhibitor efficiency. Growth factor-deprived UT7 cells were pre-treated for 15 min with 50 μM LLnL (L), 1 μM MLN4924 (M) or 10 μM Pyr41 (P41). Cells were then stimulated for 30 min with 10 units/ml Epo and cell extracts were analysed by western blot. ER-1 corresponds to the maturation form of the EpoR that is localized in the endoplasmic reticulum and ER-2 corresponds to the mature form present at the cell surface that is degraded by proteasome after Epo-stimulation [20]. (**C)** Pyr41 an ubiquitin-activating enzyme (E1) inhibitor did not modify Pim2 stability. UT7 cells were treated for 1 h with 10 μM Pyr-41 and/or 50 μM LLnL or vehicle (0.1% DMSO). Cycloheximide (200 μM) or vehicle (0.1% ethanol) was added for the last 30 min of incubation and Pim2 expression was analysed by western blotting.

Since Pim2 is a very short lived protein and since the three isoforms that differ only at their N-terminus exhibit significantly different stabilities, we tested the hypothesis that the N-end rule pathway [29] could be involved in the degradation of Pim2. However, blocking type 1 and type 2 arginine/N-rule pathways using L-A, W-A, F-A dipeptides, amides forms of leucine, phenylalanine or tryptophan [30,31], tannic acid or merbromin, two arginyltranferase inhibitors [32] did not increase Pim2 stability. Moreover, bestatin methyl ester, a cell permeable inhibitor of aminopeptidases that are frequently required to expose destabilizing residues did not significantly increase the stability of Pim2 isoforms (Supplementary Figure S1).

Finally, we wondered if Pim2 could be degraded by the proteasome without ubiquitination. Indeed, previous studies suggest that this mechanism could be less uncommon than previously considered [33] and we did not observe obvious proofs of Pim2 ubiquitination in our first experiments. To test this hypothesis, we used the E1-ubiquitin ligase inhibitor Pyr41 [34]. Although Pyr41 protects Epo-activated EpoR from proteasome degradation as efficiently as MLN or LLnL (Figure 5B), Pim2 degradation was not modified by this inhibitor in the same UT7 cells (Figure 5C).

Then, we tried to detect ubiquinated forms of Pim2 using sensitive methods. To do that, cells were treated with proteasome inhibitors and cell lysates were analysed by western blotting using anti-Pim2 antibodies. Despite strong overexposure of the blots, we did not detect Pim2 forms of higher molecular masses than those of the three isoforms. To rule out that Pim2 could be quickly de-ubiquitinated, we treated UT-7 cells with proteasome inhibitors in the presence of inhibitors of DUBs. Three DUB inhibitors were used: *N*-ethylmaleimide, a thiol alkylating reagent, PR619 and WP1130, two wide-spectrum DUB inhibitors. Altogether, DUB inhibition did not reveal ubiquitination forms of Pim2 even when proteasome degradation was also inhibited (Figure 6A). Similar results were observed in myeloma cells (result not shown). We probed anti-ubiquitin immunoprecipitates from bortezomib-treated myeloma cells with Pim2 antibodies. Pim2 was not detected in the immunoprecipitates (Figure 6B, left) although high amounts of ubiquitinated proteins were detected when immunoprecipitates were re-probed with anti-ubiquitin antibodies (Figure 6B, right). We performed the reverse experiment. When Pim2 immunoprecipitates were probed with anti-ubiquitin antibodies, no ubiquitinated form of Pim2 was detected (Figure 6C). Altogether, these results strongly suggested that Pim2 could be degraded by the proteasome without ubiquitination.

**Figure 6:**
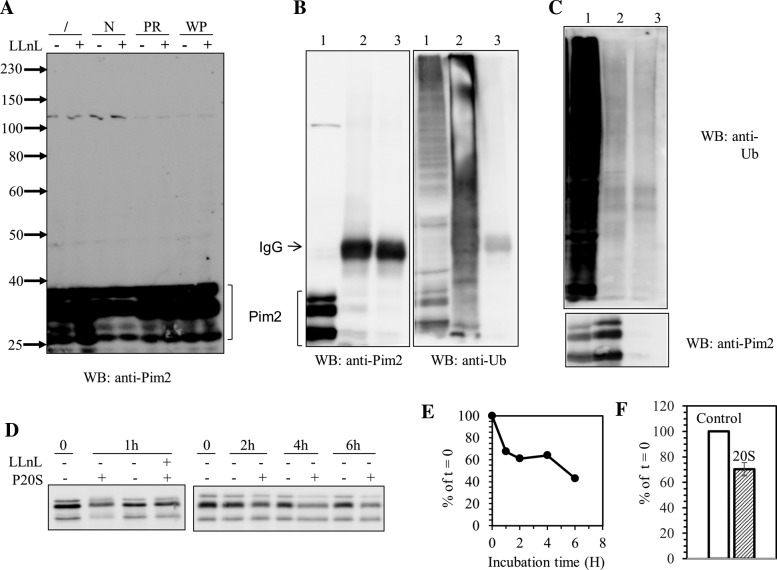
Analysis of Pim2 ubiquitination (**A)** UT7 cells were incubated for 1 h without (/) or with 50 μM LLnL, 2 mM *N*-ethylmaleimide (N), 50 μM PR-619 (PR) or 10 μM WP1130 (WP). Cell extracts were analysed by western blot using Pim2 antibodies. Blots were strongly overexposed to improve the detection of minor bands. (**B**) Extracts from AMO1 cells were immunoprecipitated with anti-ubiquitin rabbit antibodies and successively blotted with anti-Pim2 and anti-ubiquitin mouse antibodies. 1: whole cell lysate, 2: anti-ubiquitin antibody immunoprecipitate, 3: control immunoprecipitate (normal rabbit serum). (**C**) Pim2 was immunoprecipitated from AMO1 myeloma cells pre-incubated for 1 h with 50 μM LLnL. Immunoprecipitates were successively probed with anti-ubiquitin (Ub) and anti-Pim2 antibodies. 1: whole cell lysate, 2: anti-Pim2 immunoprecipitate, 3: control immunoprecipitate (the pre-immune serum corresponding to the anti-Pim2 serum was used as control serum). (**D**) Pim2 was purified from bortezomib-treated myeloma cells using ion exchange and size exclusion chromatography to test its direct degradation by 20S proteasome. Left panel: Partially-purified Pim2 was incubated with purified 20S proteasome particles for 1 h at 37°C. Immediately boiled extracts (0), extracts incubated without proteasome and extracts incubated with proteasome and LLnL were used as controls. Right panel: the same extracts were incubated for the indicated times at 37°C with or without 20S proteasome. (**E**) Densitometric scanning quantification of the experiment presented in (**D**). (**F**) Partially purified Pim2 was incubated for 2 h without (control) or with purified 20S proteasome (20S). Pim2 degradation was analysed by western blot. Densitometric scanning of the blots was performed and Pim2 degradation by 20S proteasome is expressed relative to the incubation without proteasome. The graph presents the mean ± S.D. of three independent experiments.

To test this hypothesis, we partially purified Pim2 from bortezomib-treated myeloma cells using ion exchange and size exclusion chromatography. Using these methods, Pim2 was not submitted to harsh conditions as those required for elution from antibodies and its structure is less susceptible to be modified. Moreover, the size exclusion chromatography step excludes potentially ubiquitinated forms that could have been missed in our previous experiments. When Pim2-enriched preparations were incubated for 1 h with purified 20S proteasome particles, we observed a partial degradation of the three Pim2 isoforms that was inhibited by LLnL (Figure 6D, left panel). Increasing the incubation time up to 6 h increased the amount of Pim2 degraded by purified 20S proteasome [Figures 6D (right panel) and 6E]. A partial degradation of Pim2 by purified 20S proteasome particles was constantly observed in three independent experiments (Figure 6F). These results strongly suggest that Pim2 could indeed be degraded by the proteasome without ubiquitination.

### Pim2 inhibition improves proteasome inhibitor efficiency in multiple myeloma

In all cells, proteasome inhibition led to a rapid and strong accumulation of the three Pim2 isoforms. We tested whether accumulated Pim2 isoforms were catalytically active. RPMI 8226 myeloma cells were treated for 1 h with bortezomib and Pim2 was immunoprecipitated from cell extracts. A kinase assay was performed using immunoprecipitates to evaluate Pim2 activity in control cells and in cells treated with proteasome inhibitors. The pro-apoptotic BH3-only protein Bad, a validated Pim2 substrate [35], was used as substrate in this kinase assay. Bad phosphorylation was strongly increased in Pim2 immunoprecipitates from bortezomib-treated cells (Figure 7A).

**Figure 7:**
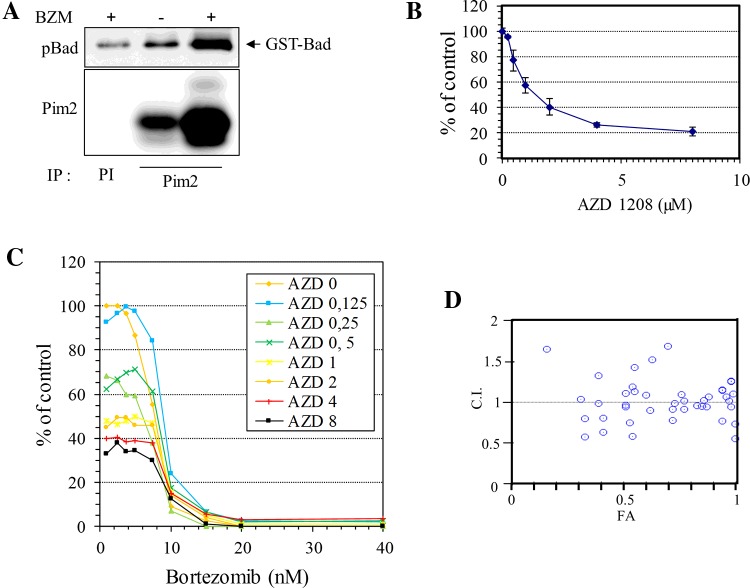
Pim kinase inhibitors inhibit myeloma cell growth (**A**) Bortezomib induces the accumulation of active Pim2 in myeloma cells. RPMI8266 cells were treated for 1 h with 100 nM bortezomib (BZM) and solubilized. Pim2 was precipitated using pre-immune (PI) or anti-Pim2 serum. Kinase activity of immunoprecipitates was tested using recombinant GST–Bad as substrate. Bad phosphorylation was detected by western blot using anti-phospho-Bad (S112) on supernatants of the kinase assay. Immunoprecipitated Pim2 was then eluted from the beads and analysed by western blot to control immunoprecipitation efficiency. (**B**) Sensitivity of RPMI 8226 cells to AZD 1208. Cells were incubated for 48 h with AZD 1208 and cell proliferation was quantified using UptiBlue, a fluorescent analogue of MTT. (**C**) Sensitivity of RPMI 8226 cells to bortezomib and AZD1208. Cells were incubated for 48 h with the indicated concentrations of AZD 1208 and bortezomib. Cell proliferation was quantified using Uptiblue (**D**) Analysis using the Compusyn software of the data presented in (**C**) Abbreviations: C.I., combination index; FA, fraction affected.

Inhibiting Pim kinase activity impaired the growth of myeloma cells (Figure 7B). Our results confirm that Pim kinases play an important role in the survival and proliferation of myeloma cells [5,7,36]. Since proteasome inhibitors that are frequently used to treat myeloma induce the accumulation of catalytically-active Pim2 protein in myeloma cells, we examined the effect of both inhibitors on myeloma cell lines. A strong inhibition of cell proliferation and survival was observed in cells treated with these inhibitors (Figure 7C). These results were analysed using the Chou and Talalay method [24] showing that AZD 1208 and bortezomib presented additive effects to inhibit myeloma cell growth (Figure 7D). We conclude that a combined treatment by these two inhibitors can be promising in myeloma.

## DISCUSSION

In the present work, we studied the mechanisms that control Pim2 accumulation in transformed haematopoietic cells. Three Pim2 isoforms were detected and their relative amounts were remarkably similar in all tested cell lines. Isoform 2 is both the most abundant and the most rapidly degraded Pim2 isoform. Consequently, more Pim2 proteins are produced from the second initiation site despite its CUG initiation codon than from the two other translation initiation sites. In some experiments, a fourth band migrating between isoforms 2 and 3 was detected, especially in UT7 cell extracts. This band is most probably a Pim2 protein since it follows both the same degradation kinetics and the accumulation in the presence of proteasome inhibitors than Pim2 isoforms 2 and 3 (Figures 3A and 5C). This band could correspond to a modification of isoform 2 or 3 produced *in vitro* during cell preparation since its intensity increased with increasing preparation times (Figure 3D).

Our results strongly suggest that Pim2 expression was exclusively regulated at the level of mRNA accumulation. Indeed, we did not detect any control of its mRNA translation by mTORC1 or any regulation of its degradation by Erk, mTORC1 or Akt signalling pathways. Moreover, the broad-spectrum phosphatase and kinase inhibitors okadaic acid or staurosporine did not modify Pim2 accumulation or stability in leukaemic cells (Figure 4B). Although we cannot formally exclude that Pim2 stability was regulated by phosphorylation/dephosphoryation mechanisms insensitive to these inhibitors, our results suggest that Pim2 stability is probably constitutive and not regulated by signalling mechanisms. This result is in accordance with our observation that Pim2 protein levels are strongly related to their Pim2 mRNA levels in all tested haematopoietic cells (E. Lestang, P. Mayeux, unpublished data). At the mRNA accumulation level, Pim2 expression was controlled by Epo in the UT7 cell line and we showed that this regulation involved STAT5. In UT7 cells, both shRNA targeting either STAT5A or STAT5B decreased Pim2 expression and shRNA targeting both STAT5 isoforms were more efficient than shRNA targeting a single STAT5 isoform, showing that both STAT5 isoforms are able to regulate Pim2 mRNA accumulation. These results extend those recently published for the MOLM14 and MV4.11 cell lines [37] and demonstrate that STAT5 controls Pim2 mRNA expression in AML cells. Interestingly, cytokines that activate STAT5 like Epo induced Pim2 expression and sustained long-term UT7 cell proliferation. In contrast, SCF that did not activate STAT5 only briefly stimulated UT7 cell proliferation. It has been reported that Pim2 was able to complement wild-type Flt3 to induce murine 32D cell proliferation [38]. We tested whether forced Pim2 expression could complement SCF stimulation to sustain UT7 cell growth. To do this, we transfected UT7 cells with an expression vector allowing the constitutive expression of Pim2. Although Pim2-transfected UT7 cells slightly increased their proliferation rate in the presence of SCF, their growth did not reach the level of Epo-stimulated UT7 cells (result not shown).

mTORC1 controls the translation of mRNA with long structured 5′-UTR mainly through inactivation of the 4E-BP1 inhibitor of translation initiation. Pim2 expression was not decreased by direct inhibition of mTORC1 that indeed induced the dephosphorylation of 4E-BP1. Moreover, Pim2 expression was not increased in UT7 cells stimulated by SCF compared with growth factor-deprived cells (Figure 2A). Akt, which in turn controls mTORC1 activity, is strongly activated by SCF in UT7 cells. Since Pim2 mRNA levels were roughly similar in growth factor-deprived and in SCF-stimulated cells (Figure 2C), these data confirms that Pim2 translation is not controlled by mTORC1.

The activity of Pim2 is constitutive and its degradation does not seem to be substantially regulated. The degradation of the three Pim2 isoforms is very rapid and Pim2 is probably one of the most unstable cellular proteins. The half-life of the three Pim2 isoforms was very similar in all haematopoietic cells. Nevertheless, isoform 1 appears significantly more stable than the two shorter isoforms, thus showing that the N-terminus part modulates Pim2 stability. We did not observe a different sub-cellular localization of isoform 1 that could explain its increased stability. This suggested that this difference of stability could be an intrinsic property of the isoforms. Involvement of the N-end rule pathway machinery in the ubiquitination of Pim2 and the control of its degradation could be an attractive hypothesis to explain the different stabilities of Pim2 isoforms [29]. However, inhibition of this ubiquitination pathway did not increase Pim2 stability. In agreement with this result, we did not detect Pim2 ubiquitination even when its degradation was blocked by proteasome inhibitors. Ubiquitinated proteins can sometime be difficult to observe because they are rapidly de-ubiquitinated. Therefore blocking DUB can facilitate their detection [39,40]. However, treating cells with both proteasome and DUB inhibitors did not allow the detection of ubiquitinated forms of Pim2. These results are hardly compatible with the hypothesis that Pim2 is degraded after ubiquitination. Indeed, because of the very short half-life of Pim2 isoforms, blocking both their degradation and their de-ubiquitination should have led to the strong accumulation of their ubiquitinated forms. Inability of our antibodies to recognize the ubiquinated forms of Pim2 is unlikely since two different antibodies were used for immunoprecipitation and for western blot. Moreover, the E1-ubiquitin ligase inhibitor Pyr41 did not slow down the degradation of Pim2 whereas, in the same cells, it was as efficient as proteasome inhibitors to reduce the degradation of the activated EpoR that is degraded by the proteasome after ubiquitination with similar kinetics than Pim2 [20]. Overall, our data show that the bulk of Pim2 could be degraded by the proteasome without ubiquitination. Several proteins including proto-oncogenes have been reported to be degraded likewise [26]. Proteins degraded by proteasomes without ubiquitination have frequently long disordered regions [41]. Structural requirement for proteasome recognition and degradation in the absence of ubiquitin have been thoroughly studied for thymidylate synthase, apomyoglobin and p53 [42–44]. In all these cases, ubiquitin-independent degradation requires the presence of unfolded regions even though these regions can be surrounded by structured regions. The unstructured regions of thymydylate synthase and of p53 that are responsible for their ubiquitin-independent degradation correspond to the N-terminal part of the molecules [42,45]. Interestingly, the first 30 amino acids of Pim2 corresponding to the region before the kinase domain appear to be unstructured in Pim2 isoform 3 [9]. Analysing isoforms 1 and 2 that have not been crystallized and possess longer N-terminal parts using the PSIPRED v3.3 / DISOPREP2 software (http://bioinf.cs.ucl.ac.uk/psipred/) shows that these extended domains are completely disordered (Supplementary Figure S2). Moreover, this analysis suggests that the C-terminal part of the molecule could also be disordered although to a lesser extent. Thus, we suggest that the disordered N-terminus domains of Pim2 isoforms are directly recognized by the proteasome although with different efficiencies that could account for the differences of stability among the three isoforms. To sustain this hypothesis, we incubated partially purified Pim2 molecules with purified 20S proteasome. For this experiment, we took care not to expose Pim2 isoforms to denaturing conditions that could favour their degradation by artificially creating disordered regions. Our results show that indeed Pim2 can be a direct substrate of 20S proteasome.

It is currently unknown whether other Pim kinases could also be degraded by a similar mechanism involving proteasomes without prior requirement for ubiquitination. Indeed, although proteasome-mediated degradation of Pim kinases is well established for the three Pim kinases, no E3-ubiquitin ligase that mediates ubiquitination has been identified to our knowledge for any of them. Ubiquitination of the different Pim kinases has been sometime reported. In many cases these experiments have been performed using transfected cells that expressed high amounts of the exogenous kinases. Nevertheless, in some cases, ubiquitination of the endogenous forms of Pim kinases has been detected (for example, see study by Chen et al. [46] regarding Pim1 ubiquitination), suggesting that part of the kinase could be also degraded by a classical ubiquitin-dependent proteasome process. Proteasome degradation by both ubiquitin-dependent and -independent mechanisms has been previously described for proteins such as Mcl1 or p53 [47,48] and cannot be excluded for Pim kinases. The importance of ubiquitination for Pim kinase degradation needs to be clearly established and this determination requires the identification of the E3 ligase responsible for their ubiquitination. It has been reported that Pim1 was protected from ubiquitin–proteasome degradation by a hypoxia-dependent mechanism that involves HSP90 binding [46]. The authors showed that, in contrast with Pim1, Pim2 and Pim3 were not regulated by this mechanism. Interestingly, we did not detect the association between HSP90 and Pim2 in immunoprecipitation analyses and geldanamycin did not modify Pim2 stability in AML cells (K. Adam and P. Mayeux, result not shown). This suggests that the stability of each Pim kinase could be regulated by different mechanisms. Recently, Zhang et al. [14] have reported that TCTP protein associates with the terminal domain of Pim3 and protects Pim3 from proteasome degradation [14]. Unfortunately, the reported data do not indicate whether the short specific sequence that follows the kinase domain is required for TCTP binding. Indeed, whereas these sequences are fully divergent in Pim2 and Pim3, Disoprep analysis shows that the C-terminal sequence of Pim3 is also disordered. An attractive hypothesis would be that these terminal domains contribute to direct proteasome targeting while providing means to specifically control the stability of each Pim kinase through association with different partners.

Pim2 is an important pro-survival mediator in myeloma cells, at least in part by its contribution to mTORC1 activation [5,7]. Accordingly, several Pim inhibitor molecules such as AZD 1208 or PIM447 (formerly LGH447) are currently tested in phase 1 clinical trials for multiple myeloma therapy (https://clinicaltrials.gov). Bortezomib (Velcade) and other proteasome inhibitors inhibit the growth of myeloma cells and induce their apoptosis [49]. These drugs are commonly used for myeloma therapy [50,51], although most patients relapse. Drug combinations targeting different pathways important for myeloma cell survival are currently tested [52]. Since bortezomib inhibition strongly increases the accumulation of catalytically active Pim2, it was important to test whether Pim kinase inhibitors could increase the efficiency of proteasome inhibitors to induce myeloma cell death. Although we showed that these inhibitors were not synergistic, we observed that myeloma cells were sensitive to the additive action of both proteasome and Pim kinase inhibitors for inhibition of proliferation and cell survival, thereby suggesting that their therapeutic combination may be of benefit in this disease.

## Abbreviations

A-loop: activation loop
AML: acute myeloid leukaemia
C-loop: catalytic loop
DUB: de-ubiquitinase
Epo: erythropoietin
EpoR: erythropoietin receptor
FCS: fetal calf serum
HSP: heat-shock protein
RT-qPCR: Reverse transcription quantitative polymerase chain reaction
SCF: stem cell factor
TCTP/TPT1: translationally-controlled tumour protein
